# Measuring welfare in rearing piglets: test–retest reliability of selected animal-based indicators

**DOI:** 10.1093/jas/skad162

**Published:** 2023-05-20

**Authors:** Johanna Witt, Joachim Krieter, Thore Wilder, Irena Czycholl

**Affiliations:** Institute of Animal Breeding and Husbandry, Christian-Albrechts-University, Kiel 24098, Germany; Institute of Animal Breeding and Husbandry, Christian-Albrechts-University, Kiel 24098, Germany; Institute of Animal Breeding and Husbandry, Christian-Albrechts-University, Kiel 24098, Germany; Department of Veterinary and Animal Sciences, University of Copenhagen, Frederiksberg 106 91, Denmark

**Keywords:** animal-based indicators, animal welfare assessment, rearing piglets, test–retest reliability

## Abstract

The “Welfare Quality protocols” (WQP) were developed in 2009 as objective welfare assessment tools. The WQP are based on four welfare principles: 1) “good feeding”, 2) “good housing”, 3) “good health”, and 4) “appropriate behavior”. The included WQP-indicators were developed for growing pigs and are recommended for rearing piglets, although, to the authors’ knowledge, they have not been tested in this age class. Therefore, the present study tested selected indicators from different welfare assessment protocols with regard to test–retest reliability (TRR), consistency over time, in an on-farm study on rearing pigs. This allows to investigate whether the WQP-indicators developed for growing pigs can be recommended for rearing piglets and whether the additional indicators should be included in the WQP.

In total 28 selected pen- or individual-level indicators were used by one observer to assess the animal welfare of rearing piglets on three pig farms. Per batch 40 to 125 piglets were randomly selected and individually marked to record the weekly assessments. This procedure was repeated in three consecutive batches per farm and resulted in a total of 759 rearing piglets being assessed.

Spearman’s rank correlation co-efficient (RS), intraclass correlation co-efficient (ICC), and limits of agreement (LoA) were calculated to evaluate their TRR, especially if the TRR was influenced by the group of assessed animals (batch comparisons) or the age of the assessed piglets (age class comparisons). From the 28 indicators, 12 had a very low prevalence of <1% making an assumption about their TRR meaningless. From the pen level indicators, “sneezing” achieved acceptable TRR for both comparisons and “behavioral observations” (BO) achieved in general good values (e.g., “positive social behavior”: (RS: 0.34 to 0.89; ICC: 0.00 to 0.90; LoA ϵ [−2.93; 7.41] to ϵ [−18.9; 11.5]) for both comparisons (batch, age class). The WQP-indicators of sufficient TRR, such as “tail lesions”, “lameness”, “wounds on the body”, “human–animal-relationship test” and “BO”, cannot cover the four welfare principles adequately. In particular, problems remained with the welfare principles of “good feeding”, “good housing”, and partly “good health”. However, these grievances could be overcome by including further indicators from other sources outside the WQP which have acceptable to good results for TRR in this study, such as “back posture”, “ear lesions”, “normal behavior”, and “tail posture”.

## Teaser

Teaser Text

The present study tested selected animal welfare indicators from different welfare assessment protocols, e.g., the Welfare Quality protocol for pigs, with regard to test–retest reliability (TRR), i.e., consistency over time in an on-farm study on rearing pigs.

## Introduction

In the last two decades, the welfare of farm animals has increasingly become the focus of public attention and customers have demanded transparency regarding the housing conditions of the food-producing livestock (Broom, 2010; European Commission, 2016). The growing public discussion led to the Welfare Quality research project, during which the “Welfare Quality Animal Welfare Assessment Protocols” (WQP) were developed. These are based on the four following welfare principles: 1) “good feeding”, 2) “good housing”, 3) “good health”, and 4) “appropriate behavior”. Each of these principles is defined by different criteria characterized by one or more different indicators (Botreau et al., 2007; Blokhuis, 2008). The indicators of the WQP are mainly animal-based, as these can best reflect the actual state of the animal as opposed to resource- or management-based indicators, which rather assess the risk of the husbandry environment. However, it is well-known that animal-based indicators pose a challenge with regard to feasibility, validity, and reliability (Velarde and Geers, 2007; Blokhuis et al., 2010). Most of the indicators had been tested for their feasibility, validity, and reliability in preliminary studies on growing pigs (Forkman and Keeling, 2009) before they were included in the Welfare Quality protocol (WQP). However, to the authors’ knowledge, no studies have yet been conducted on the TRR of the indicators in the age group of rearing piglets.

In Germany, the ongoing debate about welfare and transparency led to a legal change in the national law for animal protection (German designation: Tierschutzgesetz) in 2014. Since then, the owners of livestock have been legally obligated to undertake a farm self-monitoring using animal-based indicators (Anonymous, 2006a). In 2016, The Association for Technology and Structures in Agriculture (German designation: Kuratorium für Technik und Bauwesen in der Landwirtschaft e.V., KTBL) published a guideline for a German farm’s self-monitoring of pigs, which is based on the WQP. However, reliability studies on the final guideline in the rearing period are lacking. Both welfare assessment protocols do not contain any indicators specifically selected for rearing piglets. It is recommended to use the existing indicators for growing pigs in the rearing period, although reliability has not been verified for this age (Welfare Quality, 2009; KTBL, 2016). Therefore, the overall aim of the present study was to clarify whether the recommended indicators for growing pigs are suitable for use in rearing piglets, as advised in the guideline and protocol. In particular, the TRR of the indicators in rearing piglets was evaluated by an on-farm study.

Reliability describes the similarity in the results of repeated measurements on the same object. On-farm reliability is mainly defined by the interobserver and the test–retest reliability (TRR; de Passillé and Rushen, 2005). The interobserver reliability describes the extent of equal results when different observers independently assess the same animals at the same time under the same circumstances. The TRR is the agreement between assessments performed by the same observer on the same objects at different points in time. It describes the consistency of a method over time, which is an important characteristic of the assessment protocols (Martin und Bateson, 1994; Temple et al., 2013). An indicator with a good consistency over time does not react to minor changes on-farm (e.g., different batches), but major changes should be detected by the measurement tool (Plesch et al., 2010). Therefore, measurement tools with a good TRR should achieve the same results even in the presence of minor changes in the on-farm situation (Windschnurer et al., 2009).

To evaluate the TRR, i.e., the consistency of the indicators over time, two different questions were considered. The first question aimed to clarify whether the TRR between the batches of all farms differed from each other. This was intended to give an advice on whether it causes a change with regard to the overall welfare assessment of a farm when different animals are assessed on the same farms. The assumption is that these minor changes (different batches) do not influence the welfare status of the farm. The second approach analyzed the TRR between the age classes of all farms. Thereby, the visits of one age class of all farms and batches were compared to the visits of the following (older) age class, i.e., the same animals were assessed. This comparison provided information on whether the age of the piglets was significant in the implementation of the monitoring. Therefore, selected WQP-indicators and selected indicators originated from other sources (other-indicators) were used with the overall goal to find feasible, viable and reliable indicators for the animal welfare assessment of rearing piglets.

## Materials and Methods

### Ethical statement

No animal experiments were carried out. By nature of welfare assessment protocols, the indicators used should not pose harm, suffering, injury, or stress to the animals. All procedures (e.g., marking of animals, routine health checks) were carried out primarily for the farmers’ management purposes and not for scientific ones. The animals in the study were normally farmed animals and were housed conventionally or according to the EU organic scheme (Anonymous, 2007). In both cases, the animals were kept according to EU as well as national law “German Animal Welfare Act” (German designation: TierSchG; Anonymous, 2006a) and the “German Order for the Protection of Production Animals used for Farming Purposes and other Animals kept for the Production of Animal Products” (German designation: TierSchNutztV, Anonymous, 2006b).

### Data collection

Data collection was carried out on three farms with a closed system in Schleswig-Holstein, northern Germany, between October 2020 and February 2022. The farms participated on a voluntary basis and differed in production factors to increase variance. This allows to obtain a better indication of the TRR and possible influence on the indicators by the age of the animals and/or by changing animal groups (batches). The differences in production factors are shown in Table 1.

**Table 1.** Overview of the production factors of the three farms included in the study (d = days; m^2^ = square meter; *N* = numbers)

Farm	A	B	C
Production type	Organic	Conventional	Conventional
Herd size, *N* sows	50	1400	60
Suckling period, d	50	28	28
Rearing pen size, m^2^	31^1^	11.3/8.1	2.6
Group size/pen, *N*	Approx. 26	50/20^2^	10/5^2^
Space/piglet, m^2^	1.20^1^	0.22/0.40^2^	0.26/0.52^2^
Rearing pen floor	Concrete with straw	Partially slatted	Fully slatted
Outdoor arena, m^2^	13	No	No
Feeding system	By hand plus roughage	Liquid feeding	By hand

^1^Values include the outdoor arena;

^2^Two values for this cell, since the piglets were rehoused within the rearing period.

Three farms were visited by the same observer, who had been trained in the assessment of animal welfare indicators on farm by a member of the Welfare Quality Network before the study started. The study started with the random selection of a group of sows to be farrowed, ranging from 4 to 10 sows depending on the different herd sizes of the farm (Farm A: 50 sows, Farm B: 1400 sows; Farm C: 60 sows). The first visit to the animals in this study, i.e., the piglets of the randomly selected sows, took place in the week after farrowing. Due to management reasons, the piglets were individually marked on these farms, which made the animals recognizable at every visit. The following visits were usually carried out weekly (with a few exemptions due to disease, holidays, or weather conditions). For the weekly assessment, the piglets were restrained as a part of routine health checks. The piglets were crossbred (dam genetics: Large White, Danish/German/Norwegian Landrace, Danish Yorkshire; sire genetic: Pietrain).

The exact number of piglets that became part of this study depended on the number of piglets born alive from the group of sows and ranged between 40 and 120 piglets per batch (batch = time period from birth to slaughter). Three batches per farm were visited weekly over a period of 1 yr so that in total 759 piglets were assessed. Only the assessments collected in the rearing barns were included in this study. The detailed sizes of batches during the rearing period is shown in Table 2.

**Table 2.** Overview of the rearing piglets assessed per batch and the number of visits per batch within the rearing period

Farm	Batch	Number of piglets	Time period	Days rearing period	Number of visits
A	1	117	Nov 20 to Dec 20	20	2
A	2	109	Mar 21 to May 21	40	5
A	3	60	Sep 21	14	2
B	1	103	Jan 21 to Mar 21	61	8
B	2	106	Jan 21 to Mar 21	57	8
B	3	106	Jun 21 to Aug 21	48	6
C	1	40	Mar 21 to May 21	61	9
C	2	40	Mar 21 to May 21	34	6
C	3	78	Apr 21 to May 21	41	6

As mentioned, the randomly selected sow groups of the assessed piglets varied in size due to farm management. This led to different numbers of piglets per batch, especially in Farm C. The time piglets were kept in the rearing barns varied between the farms. This was due to different husbandry practices (organic: suckling period of 50 d) and fattening barn capacities on the farms. In general, the piglets of Farm A remained with the lactating sow in the farrowing barn for 3 wk. Thereafter, they were re-arranged into a group suckling barn with the lactating sow as well as two other sows and their litters (up to 36 piglets, depending on the litter size) in a community pen for approximately another 30 d. On the day 50 of life, the piglets of the organic Farm A were weaned and transferred to the piglet rearing barn in the already formed groups. This shortens the rearing period of Farm A about 3 wk compared to the conventional farms. The piglets from Farms B and C were weaned after the suckling period of 4 wk and transferred to the rearing barn. The piglets were transferred to another pen on Farms B and C within the rearing period, which resulted in two different values for “space per piglet” (compare Table 2). The rearing period ended for all farms between days 63 and 90 of life, which corresponded to a live weight of approximately 30 kg. This wide range of the end of the rearing period was due to capacities available in the fattening house. This resulted in a different number of days in the rearing barn and thereby in a different number of visits per batch as shown in Table 2. The piglets on Farms A and C were fed twice a day by hand in a long trough with dry feed according to the growth curves. The piglets on Farm B were fed several times a day by liquid feeding.

The piglets of Farms A and C had undocked tails. Those of Farm B had docked tails. There were no major changes regarding the farm’s management (e.g., weaning time) throughout the data collection.

### Protocol assessments

The weekly assessment on the individually marked piglets were based on selected health and welfare indicators from different protocols that had been recommended with regard to use in regular self-assessment to fulfill the national law requirement. In particular, the included indicators were on one side derived from the WQP for pigs—hereafter called “WQP-indicators”. On the other, they were derived from other sources, such as the German guideline for farm self-monitoring, the German literature database (Welfare Quality, 2009; KTBL, 2016; NaTiMon, 2021) and standard health checks from veterinary routine practice (Baumgartner, 2009) —hereafter called “other-indicators”. The resulting individual protocol for this study contained on the one side pen-level indicators (PIN), all originated from the WQP, of which the “behavioral observations” (BO) such as the “human–animal-relationship test” (HAR) were considered separately. On the other, the resulting protocol for this study contained animal-based individual-level indicators (IIN), partly from WQP and partly from other sources, which are described below. Hence, this is a comprehensive study of reliability of welfare indicators described in literature up to date for potential use in rearing piglets. The complete list of indicators, their source, their welfare principle, their definitions, and scoring scale are presented in Table 3. The indicators were either scored using a three-point scale (category 0 = absent, category 1 = light ­appearance, ­category 2 = strong appearance) or a two-point scale (category 0 = absent, category 1 = present). The indicators were used following the guidelines and explanations of the respective protocols/descriptions they originated from. The ­classification of the WQP-indicators according to the four welfare principles (“good feeding”, “good housing”, “good health”, “appropriate behavior”) provides an overview of the part of animal welfare for which the indicator is relevant. Detailed explanations as well as descriptions can be found in the WQP for pigs, the German guideline for farm self-monitoring, the German literature database (Welfare Quality, 2009; KTBL, 2016; NaTiMon, 2021) and standard health checks from veterinary routine practice (Baumgartner, 2009).

**Table 3.** Pen-level (PIN) and individual-level indicators (IIN) with source, welfare principle, scoring scale, and definition. Only those categories presenting presence of a welfare issue (categories 1 and 2) are shown. (KTBL = German guideline for self-monitoring; Nati = German literature database; Vet = standard veterinarian health check; WQP = Welfare Quality^®^ protocol for pigs; Baumgartner, 2009; Welfare Quality, 2009; KTBL, 2016; NaTiMon, 2021)

Indicator	Source	Welfare principle	Category	Definition
WQP-PIN^1^				
HAR^a^	WQP	Appropriate behavior	1	>60% of the piglets fleeing, facing away or huddled in corner
Huddling	WQP	Good housing	1	> 0 - ≤ 20% of resting piglets display huddling behavior (pig lying with >50% of its body on top of another pig)
			2	>20% of resting piglets display huddling behavior
Panting	WQP	Good housing	1	> 0 - ≤ 20% of piglets are observed panting (rapid breath in short gasp)
			2	>20 of piglets are observed panting
Shivering	WQP	Good housing	1	> 0 - ≤ 20% of piglets are observed shivering (vibration of any body part)
			2	>20% of piglets are observed shivering
Scouring	WQP	Good health	1	Some liquid manure in the pen
			2	All feces visible inside pen are liquid
Coughing	WQP	Good health	*N*	Number of coughs per pen
Sneezing	WQP	Good health	*N*	Number of sneezes per pen
Positive social behavior^3^	WQP	Appropriate behavior	%	Sniffing, nosing, licking, and moving gently away from the animal without an aggressive or flight reaction from this individual
Negative social behavior ^3^	WQP	Appropriate behavior	%	Aggressive behavior or any behavior with a response from the disturbed animal or any tail in mouth behavior
Pen investigation^3^	WQP	Appropriate behavior	%	Sniffing, nosing, licking all features of pen
Use of enrichment material^3^	WQP	Appropriate behavior	%	Exploration towards straw and other suitable enrichment material
WQP-IIN^2^^,^^b^				
Body condition	WQP	Good feeding	1	Thin: visible spine, hip, pin bones
Bursitis	WQP	Good housing	1	One/several small bursae on the same leg or one large bursa
			2	Several large bursae on the same leg or one extremely large or eroded bursa
Lameness	WQP	Good health	1	Severely lame, weight-bearing on affected limb
			2	No weight-bearing on one limb or unable to walk
Pumping	WQP	Good health	1	Evidence of labored breathing
Rectal prolapse	WQP	Good health	1	Evidence of rectal prolapse
Ruptures/hernias	WQP	Good health	1	Small hernia/rupture
			2	Hernia/rupture touching the floor or with bleeding lesion
Skin condition	WQP	Good health	1	> 0 - ≤ 10% has abnormal color or texture
			2	>10% of skin has abnormal color or texture
Tail lesions	WQP	Good health	1	Visible lesions
Twisted snout	WQP	Good health	1	Evidence of twisted snout
Wounds on the body	WQP	Good health	1	>4 - <10 lesions on one or more zones on the body
			2	≥10 lesions on two zones or one zone > 15 lesions
Other-IIN ^2^^,^^c^				
Abdominal wall	Vet	No principle^4^	1	Abnormal (tense)
Back posture	Vet	No principle^4^	1	Abnormal (arched back)
Claw alterations	KTBL	No principle^4^	1	Evidence of alterations (injured, bleeding erosion (side wall), cracks (heel, sole, sole/heel junction, side wall), panaritium)
Eye alterations	Vet	No principle^4^	1	At least one eye with alterations (swelling, discharge)
Ear lesions	KTBL	No principle^4^	1	Scab/blood/necrosis at the base or edge of the ear
Neurological disorders	NaTi	No principle^4^	1	Evidence of neurological problem (head tilt)
Normal behavior	Vet	No principle^4^	1	Abnormal behavior (lethargic)
Reddened eyes	NaTi	No principle^4^	1	At least one reddened eye (conjunctivitis)
Tail length	KTBL	No principle^4^	1	Docked tails or partial loss
			2	Full loss (blunt)
Tail posture	NaTi	No principle^4^	1	Abnormal (hanging, jammed)

^1^Evaluation at pen-level.

^2^Evaluation at individual-level;

^3^Categories of the BO;

^4^No principle yet associated;

^a^Human-animal-relationship test;

^b^Indicators originated from the WQP;

^c^Indicators originated from other sources.

#### Pen-level indicators.

The weekly visits were scheduled in the morning after the staff had already carried out an animal check in the morning. The assessments of the PIN were carried out at the beginning of each visit. The first step was to watch the pens and count how many piglets were lying, “huddling”, “panting”, or “shivering”. After that, the pens were entered by the observer and the “HAR” was carried out. At the same time, the pen was checked for any liquid manure (“scouring”). After making the animals stand up, the number of “coughs” and the number of “sneezes” per pen occurring were recorded for 5 min. For further calculation, the number of “coughs”/“sneezes” in the 5 min was divided by the number of animals under observation.

#### Human–animal-relationship test.

After the assessment of the PIN, the “HAR” (pen-level) was carried out to evaluate the reaction of the piglets towards the observer. Therefore the corresponding pens were entered. The observer first walked clockwise along the pen wall (inside the pen) and then stood still in the middle of the pen for 30 s. Then, the observer walked counter clockwise along the pen wall and analyzed whether the piglets showed a panic response, e.g. fleeing or huddling in the corner. There was no physical interaction or talking to the animals while the observer performed the test. For further analysis of the “HAR”, the percentage of pens with a panic response from the total observed pens per farm was taken into account.

#### Behavioral observations.

The “BO” (pen-level) followed after counting the number of “coughs” and “sneezes”. Thereby, the piglets had 5 min time to calm down after the animals had been encouraged to stand up. Each pen was then scanned by the observer every 2 min and the piglets sorted into categories: “positive social behavior”, “negative social behavior”, “pen investigation”, “use of enrichment material”, “other active behavior”, or “resting”. A total of five scans were performed per pen. For further analysis, the “BO” was expressed as a percentage of the total active behavior, which was all behavior, except resting.

#### Individual-level indicators.

All individually marked piglets were scored from both sides for a variety of IIN, e.g., “bursitis”, “lameness”, and “wounds on the body” (Table 3).

### Statistical analysis

Data were processed first in Microsoft Excel (Microsoft Corporation, 2016), then in the statistical software SAS 9.4 (SAS Institute Inc., 2017). Eligibility checks were carried out before further analysis to rule out any potential transmission errors. In order to evaluate TRR, i.e., the consistency over time of the welfare indicators, the comparability between the batches and the comparability of the age classes were determined. The TRR of the three batches assessed on each farm was compared to investigate whether the TRR had been influenced by the group of animals assessed. To also evaluate a possible influence of the age of the animals on the TRR, the weekly assessment visits were classified into nine age classes, and one age class was always compared to the following age class (the visit in the following week). For both comparisons, the different categories (0, 1, and, if applicable, 2) of each indicator were treated as an independent variable and analyzed as percentages of animals assigned to the corresponding category per visit (fictive example for clarification: visit week 1: “lameness” category 0: 98% of the assessed animals, “lameness” category 1: 1.5% of the assessed animals, “lameness” category 2: 0.5% of the assessed animals). The percentages achieved in the two consecutive visits were then compared with regard to TRR. For the evaluation of the TRR of the two comparisons (batch, age class), a combination of different reliability and agreement parameters was calculated using the statistical software SAS 9.4 (SAS Institute Inc., 2017) as described below. This combination of indicators is commonly advised and has been used in comparable reliability studies (de Vet et al., 2006; Czycholl et al., 2016; Can et al., 2017; Friedrich et al. 2019a, 2019b). Interpretation of values was carried out with regard to existing literature to ensure comparability (Temple et al., 2013; Czycholl et al., 2016; Friedrich et al. 2019a, 2019b) and to general recommendations concerning these parameters (Martin und Bateson, 1994; McGraw und Wong, 1996; de Vet et al., 2006).

#### Spearman’s rank correlation co-efficient (RS).

The RS is a nonparametric measure of rank correlation (Gauthier, 2001). The RS ranges between −1 and 1, whereby values closer to 1 resemble a higher correlation and a greater confidence for TRR. RS ≥ 0.40 was interpreted as acceptable reliability and RS ≥ 0.70 as good reliability.

#### Intraclass correlation co-efficient (ICC).

The ICC places the variance between study objects in proportion to the variance between study objects plus measurement error (de Vet et al., 2006). In accordance with Shrout and Fleiss (1979), a two-way model was used. The co-efficient can reach values between 0 and 1. For interpretation an ICC ≥ 0.40 was defined as acceptable and an ICC ≥ 0.70 as good reliability.

#### Limits of agreement (LoA).

 The LoA estimates the differences between two sets of measurement values and the standard deviations of these differences (Bland and Altman, 1986). The differences between the compared visits in the present study were expressed as a percentage, i.e., they range between −100 and 100. Values close to −100 reflect higher classification of the assessed animals in the second visit, whereas values towards 100 indicate that the animals were more often categorized in higher categories of the indicators in the first visit. An interval ≤ −10.0 to 10.0 implied acceptable agreement and an interval ≤ −5.00 to 5.00 implied good agreement.

## Results

To enhance readability, only those categories of the respective indicators representing presence of a welfare issue, i.e., an observation, are shown. Thus, category 0 (0 = absent) is generally not shown.

### Pen-level indicators (PIN)

The mean values in percent as well as the standard deviation of the three batches and the nine age classes for the PIN are visualized in Table 4. “Coughing” and the three-point scale indicators “panting” and “shivering” as well as “huddling” and “scouring” (both only category 2) had a prevalence of less than 1% across all batches respectively age classes. Therefore, these indicators were not evaluated further. Of the remaining PIN, “HAR” had the highest prevalence with up to 86.8% (batch 3), whereby the prevalence (panic reaction of the piglets) tended to decrease with advancing age (increasing age class). This decreasing trend in prevalence with increasing age could also be observed in “huddling” (category 1). In contrast, “sneezing” and “scouring” (category 1) indicated no clear discernible trend and had a prevalence of 0% to 1.7% and 0% to 25%, respectively across all batches and age classes.

**Table 4.** Mean percentages of pen-level-indicators in the three different batches (B) and nine age classes (A). Only those categories presenting presence of a welfare issue (categories 1 and 2) are shown. Animals sorted into behavioral categories of the “behavioral observation” (BO) expressed as a percentage of the total active behavior

Indicator	Category	B1	B2	B3	A1	A2	A3	A4	A5	A6	A7	A8	A9
Huddling	1	1.3	14.5	6.4	17.5	18.3	18.8	6.9	0.0	0.0	0.0	0.0	0.0
Scouring	1	10.0	12.5	2.1	2.1	13.3	0.0	22.2	11.1	0.0	0.0	0.0	25.0
Sneezing	%	1.7	0.2	0.2	0.6	0.8	1.7	0.7	0.7	0.7	0.3	0.7	0.5
HAR^1^	%	58.8	69.7	86.8	83.3	87.5	77.1	65.3	66.1	63.6	66.7	75.0	33.3
Positive^2^^,^^a^	%	7.2	7.3	11.3	11.7	12.7	10.4	6.2	6.2	8.0	5.5	7.4	6.2
Negative^3^^,^^a^	%	3.5	3.9	4.0	3.5	4.8	4.2	2.7	3.0	4.0	1.7	5.4	5.9
Investigation^4^^,^^a^	%	19.9	15.0	13.3	15.9	19.5	15.6	8.3	16.5	17.4	22.9	22.9	19.3
Enrichment^5^^,^^a^	%	8.5	17.7	18.1	5.9	8.4	6.6	24.5	22.1	10.7	3.6	13.7	25.0

“Coughing”, “panting”, “shivering” as well as “huddling” and “scouring” (both in category 2) had a prevalence close to 0% in all farm visits.

^1^Human-animal-relationship test;

^2^Positive social behavior;

^3^Negative social behavior;

^4^Pen investigation;

^5^Use of enrichment material;

^a^Categories of “BO”.

Table 5 includes the statistical parameters for the indicators mentioned in Table 4 without the indicators of the “BO”, which are shown in Table 6. “Scouring” (category 1) achieved acceptable to good results for the TRR especially for the ICC. In contrast, “sneezing” showed acceptable to good results for the RS but not for the ICC. “HAR”, on the other side, achieved acceptable to good results for both reliability parameters. All the indicators mentioned rarely achieved acceptable or good values for the LoA except “sneezing”.

**Table 5.** Spearman’s rank correlation co-efficient, **RS**; intraclass correlation co-efficient, **ICC**; limits of agreement, **LoA** for the pen-level indicators for the comparisons (C) of the three batches (B) and nine age classes (A) indicating poor (normal type), acceptable (italic type) and good agreement (bold type). Only those categories presenting presence of a welfare issue (categories 1 and 2) are shown

	C	Scouring^2^	Huddling^2^	Sneezes	HAR^1^
		Category 1	Category 1	*N*	Category 1
RS	B1B2	*0.67*	0.22	0.05	*0.63*
	B1B3	*0.48*	−0.11	0.02	0.33
	B2B3	0.00	−0.13	−0.35	0.33
	A1A2	0.00	**0.83**	**0.94**	0.00
	A2A3	–	0.07	*0.64*	**0.77**
	A3A4	–	*0.42*	**0.77**	**0.90**
	A4A5	**1.00**	–	**0.88**	**0.81**
	A5A6	–	–	**0.71**	**0.84**
	A6A7	–	–	0.03	**0.95**
	A7A8	–	–	**0.80**	**1.00**
	A8A9	–	–	0.16	*0.50*
ICC	B1B2	**0.91**	0.00	*0.65*	**0.89**
	B1B3	**0.77**	0.31	*0.62*	*0.61*
	B2B3	**0.72**	0.00	0.37	*0.64*
	A1A2	*0.57*	0.00	0.26	0.00
	A2A3	*0.45*	0.00	0.15	*0.57*
	A3A4	**0.76**	0.00	0.00	0.38
	A4A5	**0.76**	0.00	0.37	*0.63*
	A5A6	*0.60*	–	0.17	**0.87**
	A6A7	–	–	0.00	**0.95**
	A7A8	–	–	*0.41*	**1.00**
	A8A9	0.15	–	*0.46*	*0.64*
LoA	B1B2	−23.3 to 28.0	−58.1 to 26.8	−2.85 to 6.25	−80.3 to 74.1
	B1B3	−44.4 to 68.7	−42.4 to 33.1	−2.71 to 6.65	−100 to 72.2
	B2B3	−50.8 to 69.8	−48.2 to 74.6	**−0.43 to 0.45**	−100 to 83.2
	A1A2	−57.6 to 35.1	−35.0 to 33.4	**−0.91 to 0.51**	−100 to 100
	A2A3	−29.0 to 55.7	−66.2 to 65.4	−5.23 to 3.31	−22.2 to 43.0
	A3A4	**0.00 to 0.00**	−23.3 to 56.7	**−2.66 to 4.15**	−33.9 to 67.3
	A4A5	−32.1 to 54.3	−25.7 to 39.6	**−0.51 to 0.51**	−42.1 to 40.5
	A5A6	−32.1 to 54.3	–	**−1.22 to 1.48**	−59.1 to 48.0
	A6A7	–	–	**−1.38 to 2.41**	−28.5 to 43.5
	A7A8	–	–	**−1.40 to 0.17**	**0.00 to 0.00**
	A8A9	−98.5 to 48.5	–	**−0.50 to 0.94**	−64.7 to 100

^1^Human-animal-relationship test;

^2^Indicator with a three-point-scale, but category 2 had a prevalence close to 0 and is therefore not presented.

**Table 6.** Spearman’s rank correlation co-efficient, **RS**; intraclass correlation co-efficient, **ICC**; limits of agreement, **LoA** for the single categories of the “behavioral observations” (BO) for the comparisons (C) of the three batches (B) and nine age classes (A) indicating poor (normal type), acceptable (italic type), and good agreement (bold type)

	C	Positive^1^^,^^a^	Negative^2^^,^^a^	Investigation^3^^,^^a^	Enrichment^4^^,^^a^
RS	B1B2	0.34	0.39	**0.80**	**0.79**
	B1B3	*0.60*	0.00	*0.55*	0.25
	B2B3	*0.40*	0.26	*0.54*	0.30
	A1A2	**0.89**	0.31	**0.89**	**0.77**
	A2A3	**0.89**	0.00	**0.94**	0.37
	A3A4	*0.66*	**0.71**	0.37	*0.60*
	A4A5	*0.50*	0.33	**0.73**	**0.87**
	A5A6	**0.78**	0.02	**0.92**	*0.64*
	A6A7	**0.80**	*0.56*	0.09	0.37
	A7A8	*0.60*	0.00	**0.80**	0.00
	A8A9	0.49	0.49	**0.99**	*0.66*
ICC	B1B2	**0.81**	0.20	**0.84**	**0.97**
	B1B3	**0.86**	*0.44*	**0.75**	**0.93**
	B2B3	**0.90**	*0.48*	**0.85**	**0.98**
	A1A2	**0.89**	0.00	**0.89**	**0.76**
	A2A3	**0.82**	0.00	**0.90**	*0.51*
	A3A4	*0.51*	0.09	**0.71**	**0.70**
	A4A5	*0.56*	*0.56*	**0.82**	**0.85**
	A5A6	*0.58*	*0.63*	**0.86**	**0.92**
	A6A7	0.35	0.12	*0.61*	**0.97**
	A7A8	0.00	0.00	**0.88**	**0.99**
	A8A9	0.00	*0.47*	**0.99**	**0.97**
LoA	B1B2	*−7.95 to 6.52*	*−7.40 to 5.65*	−16.8 to 20.1	−26.7 to 23.2
	B1B3	−18.4 to 11.3	*−9.88 to 8.12*	−16.9 to 25.6	−55.9 to 40.9
	B2B3	−18.9 to 11.5	*−7.62 to 6.67*	−14.7 to 19.3	−38.1 to 26.5
	A1A2	*−8.80 to 6.68*	**−4.59 to 2.01**	−23.3 to 16.1	−12.2 to 7.27
	A2A3	*−3.58 to 8.34*	−9.61 to 10.8	−10.5 to 18.3	−13.2 to 16.6
	A3A4	*−2.93 to 7.41*	*−4.84 to 5.74*	−17.4 to 25.3	−29.8 to 21.8
	A4A5	*−8.34 to 8.34*	*−6.59 to 6.17*	−29.1 to 12.7	−29.3 to 34.2
	A5A6	*−5.09 to 3.93*	*−6.34 to 3.80*	−7.43 to 13.5	−26.0 to 32.9
	A6A7	−4.41 to 11.53	*−2.40 to 7.21*	−17.9 to 13.1	*−7.67 to 8.05*
	A7A8	*−7.15 to 0.65*	−17.1 to 7.80	−15.9 to 8.43	*−4.28 to 7.66*
	A8A9	*−6.65 to 9.74*	*−8.17 to 6.20*	**−4.24 to 4.34**	−21.9 to 7.38

^1^Positive social behavior;

^2^Negative social behavior;

^3^Pen investigation;

^4^Use of enrichment material;

^a^Categories of “BO”.

The missing values for some statistical parameters in Tables 5 and 6 resulted from increased ties or a lack of ­variance. Thus, it was not sensible to calculate the statistical parameters due to the small number of piglets (relocation to the fattening stable), especially in the later age classes.

The results of the “BO” in Table 4 are expressed as a percentage of total active behavior, excluding resting (as advised by the WQP). The values for “positive social behavior” ranged between 5.5% and 12.7%, for “negative social behavior” between 1.7% and 5.9%, for “pen investigation” between 8.3% and 22.9% and for “use of enrichment material” between 3.6% and 25.0% across all batches and age classes.

The TRR calculation for the categories of the “BO” showed overall acceptable to good results for the statistical parameters RS and ICC for both comparison approaches (batches, age classes) except for “negative social behavior” (Table 7). All other categories of “BO” achieved good values (>0.7) for the three batch comparisons for the ICC. Further, the results for the TRR of the age class comparisons were in general acceptable to good for the categories of the “BO” mentioned. In contrast, rarely good values were achieved for the LoA.

**Table 7.** Mean percentages of individual-level indicators in the different age classes (A) and batches (B). Only those categories presenting presence of a welfare issue (categories 1 and 2) are shown

Indicator	Category	B1	B2	B3	A1	A2	A3	A4	A5	A6	A7	A8	A9
Back posture	1	1.2	1.3	0.1	1.4	2.5	1.0	0.9	0.1	0.3	1.6	1.5	0.0
Claw alteration	1	1.9	0.3	1.3	0.9	1.3	0.9	0.9	1.0	1.2	1.7	1.3	2.6
Ear lesions	1	16.6	21.8	17.2	21.6	10.0	16.5	19.5	17.3	19.9	24.7	26.3	15.3
Lameness	1	1.1	0.8	1.0	1.6	1.4	0.6	1.3	1.0	1.0	0.3	0.2	0.0
Lameness	2	0.7	0.4	0.0	1.4	0.8	0.4	0.6	0.0	0.0	0.0	0.0	0.0
Normal behavior	1	0.8	1.9	0.3	1.5	1.5	0.9	0.7	0.3	0.4	2.1	3.4	0.0
Hernias	1	0.6	0.7	1.3	0.0	0.8	1.0	0.9	1.3	0.0	0.6	0.0	0.4
Skin condition	1	1.0	11.4	0.5	0.4	1.0	1.6	11.4	11.1	1.6	0.9	0.6	1.9
Tail length	1	57.0	63.4	37.3	50.6	51.1	51.5	56.3	56.3	44.8	68.1	76.6	36.7
Tail length	2	0.1	0.6	0.3	0.0	0.2	0.2	0.5	0.6	0.5	0.3	0.3	0.0
Tail lesions	1	4.2	6.2	8.8	5.0	3.5	4.9	6.1	5.6	10.4	3.4	3.6	14.4
Tail posture	1	0.5	2.4	7.7	1.0	3.5	2.0	5.1	2.6	8.1	0.0	0.0	0.0
Wounds	1	2.6	3.0	1.7	7.3	4.3	1.7	1.2	2.1	0.8	1.7	2.6	0.9
Wounds	2	0.1	4.9	0.1	0.2	0.0	0.0	0.3	10.2	0.0	0.0	0.0	0.0

“Abdominal wall”, “body condition”, “bursitis” (both categories), “eye alterations”, “hernias” (category 2), “neurological disorders”, “pumping”, “rectal prolapse”, “reddened eyes”, “skin condition” (category 2), “twisted snout” had a prevalence close to zero and are not shown.

### Individual-level indicators (IIN)

The mean values in percent as well as the standard deviation of the three batches and nine age classes for the IIN are visualized in Table 7. In principle, most IIN had a low prevalence. Therefore, they had low mean values across all batches and age classes. The two-point indicators with a prevalence close to zero were “abdominal wall”, “body condition”, “eye alterations”, “neurological disorders”, “pumping”, “rectal prolapse”, “reddened eyes” as well as “twisted snout”. For the three-point indicators, these were “bursitis” (both categories), “hernias” as well as “skin condition” (both category 2), which is why these indicators were not analyzed further and thus not presented. All remaining indicators had mean values below 5% except “ear lesions”, “skin condition”, “tail length”, “tail lesions”, “tail posture”, and “wounds on the body” (all category 1), whereby “tail length” (category 1) had the highest mean value over the three batches with 52.6%, followed by “ear lesions” with 18.5%.

The TRR calculation for the statistical parameters are presented in Table 8 for the WQP-indicators IIN and in Table 9 for the other-indicators (compare Table 3). In general, batch comparisons are of lower TRR than age class comparisons for IIN. For example, “hernias” (category 1), the values for the comparisons of the batches ranged from for RS: 0.15 to 0.37; ICC: 0.17 to 0.61; LoA ϵ [−2.18; 1.89] to ϵ [−3.90; 1.98] and for the comparisons of the age classes for RS: 0.00 to 0.85; ICC: 0.00 to 0.79; LoA ϵ [−1.29; 2.56] to ϵ [−3.79; 2.20].

**Table 8.** Spearman’s rank correlation co-efficient, **RS**; intraclass correlation co-efficient, **ICC**; limits of agreement, **LoA** for the comparisons (C) of the three batches (B) and nine age classes (A) for the individual-level indicators originated from the WQP for pigs (Welfare Quality, 2009) indicating poor (normal type), acceptable (italic type) and good agreement (bold type). Only those categories presenting presence of a welfare issue (categories 1 and 2) are shown

	C	Lameness		Hernias^1^	Skin condition^1^	Tail lesions	Wounds	
		Category 1	Category 2	Category 1	Category 1	Category 1	Category 1	Category 2
RS	B1B2	0.00	0.28	0.17	0.13	0.19	*0.66*	–
	B1B3	0.00	–	0.37	0.00	*0.43*	0.00	–
	B2B3	0.00	–	0.15	0.13	*0.44*	0.33	*0.62*
	A1A2	**1.00**	0.37	–	**0.77**	**0.99**	0.03	–
	A2A3	0.16	*0.63*	**0.72**	*0.69*	*0.49*	0.25	–
	A3A4	**0.72**	**0.77**	*0.42*	*0.63*	0.00	*0.60*	–
	A4A5	*0.61*	–	**0.85**	**1.00**	**0.73**	0.21	*0.65*
	A5A6	*0.49*	–	*0.54*	**0.85**	**0.75**	0.00	–
	A6A7	0.00	–	0.00	*0.52*	0.00	0.00	–
	A7A8	**1.00**	–	–	**1.00**	**1.00**	**1.00**	–
	A8A9	–	–	–	0.00	*0.66*	**1.00**	–
ICC	B1B2	**0.77**	*0.62*	0.17	0.13	*0.61*	*0.64*	0.04
	B1B3	0.39	0.14	0.37	*0.61*	*0.68*	0.07	0.00
	B2B3	*0.65*	*0.61*	*0.61*	*0.48*	**0.85**	0.07	0.05
	A1A2	**0.97**	*0.40*	0.27	0.19	**0.88**	0.00	0.00
	A2A3	**0.74**	*0.65*	**0.79**	0.39	**0.87**	0.00	–
	A3A4	0.22	*0.58*	0.28	0.10	*0.48*	0.00	0.01
	A4A5	0.34	*0.47*	0.00	*0.44*	*0.59*	*0.44*	0.05
	A5A6	0.00	–	0.00	0.12	*0.62*	0.09	0.00
	A6A7	0.00	–	0.00	*0.56*	0.19	*0.62*	–
	A7A8	0.00	–	0.00	**1.00**	0.00	**0.88**	–
	A8A9	0.00	–	0.15	0.00	*0.41*	*0.64*	–
LoA	B1B2	−**3.43 to 3.56**	**−3.12 to 3.53**	**−2.18 to 1.89**	−76.6 to 51.2	−27.4 to 21.1	*−6.92 to 6.49*	−49.6 to 37.9
	B1B3	**−3.59 to 3.63**	**−3.24 to 4.81**	**−3.75 to 1.63**	**−3.2 to 3.9**	−31.0 to 22.5	−8.79 to 11.7	**−0.79 to 0.60**
	B2B3	**−3.62 to 3.53**	**−1.59 to 2.69**	**−3.90 to 1.98**	−51.5 to 81.4	−23.9 to 22.1	−8.00 to 11.4	−39.5 to 52.6
	A1A2	**−0.80 to 1.21**	*−4.24 to 5.39*	**−3.79 to 2.20**	**−2.57 to 1.42**	*−6.08 to 9.11*	−6.12 to 12.3	−0.60 to 12.3
	A2A3	**−1.76 to 3.44**	**−1.60 to 2.42**	**−1.97 to 1.65**	**−2.57 to 1.37**	*−6.44 to 3.54*	*−3.97 to 9.21*	–
	A3A4	**−3.50 to 1.77**	**−2.42 to 1.56**	**−2.84 to 2.64**	−89.2 to 58.3	−21.8 to 13.4	**−2.00 to 3.03**	**−2.54 to 1.67**
	A4A5	**−3.68 to 4.29**	**−1.65 to 2.79**	**−1.42 to 0.71**	**−1.43 to 2.01**	*−6.56 to 7.51*	*−7.59 to 5.76*	−66.5 to 46.7
	A5A6	**−2.32 to 2.19**	–	**−2.07 to 2.61**	−48.8 to 68.4	−26.3 to 21.4	−9.15 to 10.1	−48.1 to 68.5
	A6A7	**−1.18 to 2.78**	–	**−1.73 to 3.45**	**−3.19 to 4.38**	−25.7 to 42.8	*−8.29 to 3.48*	–
	A7A8	**0.00 to 0.00**	–	**−1.29 to 2.56**	**0.00 to 0.00**	*−5.77 to 2.91*	*−8.07 to 2.94*	–
	A8A9	**−0.53 to 0.74**	–	**−1.50 to 0.74**	*−8.20 to 6.12*	−29.2 to 7.09	−4.98 to 10.1	–

^1^Indicator with a three-point-scale, but category 2 had a prevalence close to 0 and is therefore not presented.

**Table 9.** Spearman’s rank correlation co-efficient, **RS**; intraclass correlation co-efficient, **ICC**; limits of agreement, **LoA** for the comparisons (C) of the three batches (B) and nine age classes (A) for the individual-level indicators originated from the German guideline for farm self-monitoring, German literature database (KTBL, 2016; NaTiMon, 2021) and standard health checks from veterinary routine practice (Baumgartner, 2009) indicating poor (normal type), acceptable (italic type), and good agreement (bold type). Only those categories presenting presence of a welfare issue (categories 1 and 2) are shown

	C	Back posture	Claw alteration	Ear lesions	Normal behavior	Tail length		Tail posture
		Category 1	Category 1	Category 1	Category 1	Category 1	Category 2	Category 1
RS	B1B2	0.00	*0.44*	*0.64*	*0.49*	**0.79**	0.22	0.00
	B1B3	0.00	0.00	*0.53*	*0.65*	*0.58*	0.00	0.15
	B2B3	0.05	0.00	*0.68*	0.35	0.70	0.00	0.16
	A1A2	**0.90**	*0.56*	0.20	**1.00**	**0.89**	–	0.28
	A2A3	0.22	**0.82**	*0.49*	**0.70**	**0.85**	0.00	*0.69*
	A3A4	**0.77**	0.22	**0.83**	**0.77**	**0.94**	**1.00**	**0.90**
	A4A5	**0.75**	*0.52*	**0.91**	0.00	**0.95**	*0.61*	**0.72**
	A5A6	0.00	**1.00**	**0.93**	0.00	*0.40*	**0.72**	0.15
	A6A7	*0.56*	0.31	**0.94**	0.15	*0.46*	0.12	–
	A7A8	**1.00**	**1.00**	**1.00**	**1.00**	**0.95**	–	–
	A8A9	–	**1.00**	**0.99**	–	0.50	–	–
ICC	B1B2	0.16	**0.87**	**0.93**	*0.62*	**0.79**	0.22	0.20
	B1B3	0.00	*0.66*	**0.90**	*0.60*	**0.97**	*0.55*	*0.58*
	B2B3	*0.40*	0.00	**0.92**	*0.44*	**0.94**	*0.52*	**0.70**
	A1A2	*0.67*	0.00	0.32	**0.88**	**1.00**	0.00	*0.68*
	A2A3	*0.56*	0.00	*0.67*	**0.70**	**1.00**	0.00	*0.53*
	A3A4	0.00	0.00	**0.80**	0.18	**0.93**	*0.45*	0.05
	A4A5	0.00	*0.40*	**0.92**	0.27	**0.89**	*0.58*	0.25
	A5A6	0.11	0.32	**0.92**	0.00	**0.79**	*0.53*	*0.48*
	A6A7	0.21	0.37	**0.95**	0.00	**0.72**	0.00	0.23
	A7A8	0.21	**1.00**	**0.89**	0.00	**1.00**	*0.50*	–
	A8A9	0.00	**0.95**	**0.91**	0.00	*0.61*	0.26	–
LoA	B1B2	*−7.39 to 7.49*	*−3.35 to 5.78*	−38.1 to 27.7	*−6.67 to 3.91*	−39.6 to 53.7	**−1.41 to 1.12**	−20.6 to 16.5
	B1B3	*−4.27 to 7.55*	*−6.34 to 7.39*	−33.0 to 32.6	**−1.14 to 2.50**	−58.5 to 100	−58.5 to 100	−28.5 to 14.5
	B2B3	*−2.75 to 5.91*	**−4.20 to 2.47**	−38.2 to 41.9	*−3.74 to 8.12*	−55.9 to 83.2	**−1.64 to 1.65**	−30.0 to 20.6
	A1A2	*−5.48 to 3.30*	**−2.74 to 1.90**	−15.9 to 39.1	**−0.02 to 0.01**	**−2.42 to 1.58**	**−1.26 to 0.82**	−10.4 to 5.44
	A2A3	*−6.72 to 9.69*	**−1.59 to 2.42**	−32.5 to 19.5	**−1.48 to 2.71**	**−2.89 to 1.90**	**−1.34 to 1.46**	*−2.49 to 5.38*
	A3A4	**−1.42 to 2.57**	**−3.68 to 3.36**	−31.0 to 21.1	**−0.92 to 0.56**	**−0.85 to 1.27**	**0.00 to 0.00**	−30.6 to 20.0
	A4A5	**−2.33 to 3.86**	**−2.42 to 2.16**	−7.90 to 12.4	**−3.59 to 4.41**	**−0.89 to 0.87**	**−1.38 to 1.10**	−12.2 to 17.2
	A5A6	**−1.21 to 0.97**	**−0.51 to 0.72**	−27.3 to 18.8	**−2.12 to 2.04**	−96.4 to 95.5	**−1.56 to 1.58**	−28.1 to 20.7
	A6A7	**−4.53 to 2.31**	*−6.46 to 4.08*	−10.7 to 9.44	**−3.86 to 0.03**	−95.8 to 62.9	**−1.36 to 1.48**	−21.1 to 40.0
	A7A8	**−1.60 to 0.81**	**0.00 to 0.00**	−17.9 to 7.25	*−9.61 to 4.85*	**−3.77 to 0.58**	**−0.64 to 1.28**	–
	A8A9	*−3.03 to 5.47*	**−3.36 to 1.66**	−9.78 to 20.3	−4.70 to 11.1	−62.5 to 100	**−0.74 to 1.50**	–

## Discussion

### Test–retest reliability assessment

This study tested different animal welfare indicators for their TRR, especially with regard to a possible influence of the age of the assessed piglets as well as different animal groups (batches). The results varied widely depending on the indicator. Nevertheless, to cover all different aspects of welfare (and therefore the four welfare principles), a number of different indicators need to be included in welfare assessment ­protocols. There are different reasons that can lead to a low TRR (Temple et al., 2013). However, distinguishing between the different reasons was not the aim of the present study.

As general limitation of this study, it should be born in mind that the farms participated on a voluntary basis and that there were no known health problems on these farms. In addition, all farms are located in the same region with similar climatic conditions. Hence, the assumptions made are based on this and generalization to e.g., countries with differing climate may be problematic. Moreover, the included welfare related indicators based on the existing literature and existing recommendations may not be comprising all welfare indicators potentially available, especially as research on applicable welfare indicators is an on-going process.

For statistical analysis, a combination of reliability (RS, ICC) and agreement (LoA) parameters was used. De Vet et al. (2006) advised the use of several parameters for the evaluation of reliability to compensate for disadvantages as the interpretation of only one parameter can result in misinterpretation. Moreover, this combination of parameters has been used for other studies concerning the reliability of welfare assessment protocols and thus further ensures comparability to literature (e.g., Temple et al., 2013; Czycholl et al., 2016; Can et al., 2017; Friedrich et al. 2019a, 2019b). According to Wirtz and Caspar (2002), when interpreting the reliability parameters (RS, ICC), it is important to note that they depend on the total variance of the study objects, i.e., if variance among study objects is low, reliability may be underestimated. To indicate acceptable TRR, the statistical parameters should reach the values of acceptability (RS, ICC: ≥ 0.40; LoA: ≤ −10.0 to 10.0). But if variance strongly affected the explanatory power of the reliability parameters, reliability can still be interpreted as sufficient in some cases even if the reliability parameters are not acceptable (but agreement is good). If the opposite effects occur—namely acceptable reliability values without acceptable agreement values—an existing relationship can still be assumed and conclusions can be drawn between the farm visits. Although in this case, no exact agreement is present, which may limit the application of those indicators for some purposes; the order and thus the ranks of the animals remain the same, leading to good values for the reliability parameters (Czycholl et al., 2018). Hence, for ­purposes in which ­specifically the ranking of e.g., farms would be important, these indicators may still be useful.

### Pen-level indicators

Considering the prevalence of the selected indicators in this study, it is not possible to give a conclusive assessment for the following PIN: “coughing”, “panting”, “shivering” as well as “huddling” and “scouring” (both category 2). It might be that these indicators are not relevant in this phase of life (rearing period) in well-managed farms in regions without extreme climatic conditions.

The lack of variance between the farm visits made it insensible to calculate RS for “huddling” (category 1), especially in the later age groups. However, the other statistical parameters indicate low TRR.

Further, the prevalence is low for “sneezing” with mean values ranging from 0.2% to 1.7% but in a similar range to the study by Friedrich et al. (2019b) with suckling piglets and slightly higher values compared to the study by van Staaveren et al. (2018) with rearing piglets. The acceptable to good TRR detected in this study is likewise similar to the results of Friedrich et al. (2019b). This is in contrast to Temple et al. (2013), who detected poor TRR for “sneezing” and at the same time much higher mean values with 19.7% in growing pigs. The large differences can possibly be explained by different climatic conditions and a higher age of the animals. In addition, Temple et al. (2013) visited 15 different farms, which significantly increased the variance of the indicator “sneezing”, as determined by the high standard deviation.

The high prevalence and high variance between farms for “scouring” (category 1) compared to other studies should be taken into account when interpreting the present results ­(Temple et al., 2013; van Staaveren et al., 2018; Friedrich et al., 2019b). This is due to the fact that the outdoor arena of the organic farm often implied a case of “scouring”, according to the definition of the indicator, if it had previously rained a lot. Therefore, the question of the validity of the indicator arises, especially in pens with an outdoor arena. However, despite the suggestion that precipitation influences the results, good TRR was achieved in this study.

In summary, only “sneezing” and “scouring” (category 1) of the PIN achieved good results for the statistical parameters, but the latter indicator was influenced by the weather conditions in the outdoor arena.

### Human–animal-relationship test (HAR)

The prevalence of the flight reaction decreased with increasing weight and age of the piglets. This decreasing tendency explains the results of acceptable to good results for the reliability parameters (RS, ICC), while no exact agreement was achieved, as shown by the LoA. The effect of the age was also recognized in the studies by Czycholl et al. (2016) and Mieloch et al. (2020) with growing pigs. It should be noted that unlike the studies mentioned, the piglets were visited weekly in this study. It can be assumed that the animals became used to the observer’s weekly visits and no longer showed any panic reactions. This assumption is supported by the conclusion of de Passillé and Rushen (2005) that the “HAR” may be too sensitive and susceptible to minor changes. It is therefore not recommended to carry out the test on very young piglets, which are very anxious due to the change of pen and the new environment. The batch comparisons, which correspond more to the period of a self-assessment, achieve in general acceptable results for RS and ICC. This is similar to the results of Temple et al. (2013). However, overall, TRR is lower than in the age class comparisons, thereby ­revealing problems in the assessment of different groups of animals (batches).

### Behavioral observations

The recorded prevalences were in similar ranges to the prevalences from studies with growing pigs (Temple et al., 2011; Czycholl et al., 2016). Only an increased tendency for the category “use of enrichment material” could be determined, which can be explained by the straw and the feeding racks in the pens, classified as enrichment material. Moreover, it is known that the playing behavior, which includes the use of enrichment material, increases in the first 6 wk of life and then declines to lower levels by week 14 of life (Brown et al., 2015). The statistical parameters achieved overall acceptable to good results for the categories “positive social behavior”, “pen investigation”, and “use of enrichment material” for all comparisons of the batches, comparable to the results of Czycholl et al. (2016) and Friedrich et al. (2019a). In contrast, Temple et al. (2011) detected low TRR in growing pigs for all categories of the BO. According to Temple et al. (2011), the cause for the low TRR in their study was the improvement of experience in the assessment of the observer during the data collection, i.e., this result was caused by the experimental setup and is therefore not directly comparable to the studies of Czycholl et al. (2016), Friedrich et al. (2019a), and those of the present study. In the present study, again, the results of the comparisons from the age classes achieved better results for the statistical parameters than the comparisons of the batches. The comparison of the later age classes sometimes showed opposite results, because the total number of animals decreased (relocation to the fattening stable) and therefore there was more space in the pens. This resulted, for ­example, in less social behavior because the piglets were able to keep their individual space (i.e., real differences). An exception to the general acceptable to good TRR of the “BO” in this study was the category “negative social behavior”. The low TRR for the category “negative social behavior” could be explained by the lower variance from the data and by real differences between the visits. For example, the observer sometimes recorded increased “negative social behavior” during a tail biting outbreak.

### Individual-level indicators selected from the WQP

Considering the prevalence from the selected indicators sourced from the WQP (WQP-indicators), it is not possible to give a conclusive assessment for the following IIN: *“*abdominal wall”, “body condition”, “bursitis”, “pumping”, “rectal prolapse”, “twisted snout” as well as “hernias”, “lameness”, and “skin condition” (all category 2). The reasons (e.g., climatic conditions, health status of the farms, age of the pigs) that led to the low prevalence of the aforementioned IIN were probably the same as those already discussed for PIN. Van Staaveren et al. (2018) recorded similar prevalence for “hernias”, “rectal prolapse” as well as “twisted snouts” but slightly higher values for “body condition” (4.4%) at the beginning of the rearing period and higher values for “bursitis” (3.9%) at the end of the rearing period. This difference can probably be explained by the fact that in contrast to the study of van Staaveren, in the present study, small and thin piglets stayed longer in the farrowing unit (i.e., were not found in the rearing period which was solely assessed). Czycholl et al. (2016) noted a prevalence of 30.0% for “bursitis” (category 1) in growing pigs and found a poor TRR for “bursitis”. The authors justified this with the fact that it was difficult to assess due to insufficient light in the pens and moving animals. The difference in prevalence for rearing piglets and growing pigs suggests that “bursitis” at a young age is not a major problem, but develops at a later age.

The remaining WQP-indicators with higher prevalence (in category 1) are “wounds on the body”, “lameness”, “skin condition”, “hernias”, and “tail lesions”. In general, the prevalence in category 2 was very low (for indicators with a ­three-point scale) and thus, again, statistical parameters were not calculated.

An exceptional value of 10% was recorded for “wounds on the body” in age class 5. This was probably caused by the fact that eight pen partitions of one compartment were not closed correctly during batch 2 from Farm C and the 40 animals had mixed overnight, which led to rank fights. This circumstance fulfills the condition “real differences” (Temple et al., 2013). In general, the impact of rank fighting is visible for “wounds on the body” as the prevalence decreases in older piglets, i.e., the age classes. In newly arranged groups, fights occur to establish a rank order (Meese and Ewbank, 1973). These rank fights could be the reason for a low TRR for the age class comparisons, because there were real differences between the following visits. The comparisons of the batches partially achieved acceptable results for the statistical parameters. Similar TRR studies have achieved acceptable to good results for “wounds on the body” (Temple et al., 2013; Czycholl et al., 2016; Friedrich et al., 2019b).

The present study revealed acceptable to good results for “lameness”, except for the statistical parameter RS within the batch comparisons, which indicates ties in the data and again problems with the evaluation of different animal groups in different batches. Likewise, Czycholl et al. (2016) obtained acceptable to good results for the TRR, but they did not use the RS and ICC as parameters. In contrast Temple et al. (2013) and Friedrich et al. (2019b) found poor TRR for “lameness”. Friedrich et al. (2019b) interpreted that it was difficult to identify lame animals when the piglets moved as a group. They recommended an individual assessment, which was carried out in the present study and obviously improved reliability results.

Acceptable results were achieved for “skin condition”, which stands in accordance with the results of Friedrich et al. (2019a; in sows) and Czycholl et al. (2016; in growing pigs). In contrast, Temple et al. (2013) detected poor TRR results for growing pigs and argued that “skin condition” has unpredictable variations over time and affects farms sporadically. The conditions in this study differed in that the piglets were scored weekly and skin changes were sometimes visible for longer. Moreover, sunburn occurred regularly on the organic farm at certain times of the year.

Further, the indicator “hernias” achieved acceptable TRR overall in the present study. The lower TRR than in the study by Czycholl et al. (2016) for growing pigs is related to the age of the piglets, which influences the size of the hernias. In addition, small hernias can be overlooked more easily.

“Tail lesions” achieved in general acceptable to good TRR for both comparisons in the present study. This confirms the results of Czycholl et al. (2016) with good TRR for “tail lesions” in growing pigs. In contrast, Temple et al. (2013) determined a low TRR for “tail lesions” and argued that indicators with a high intrafarm variability might not be detected by the sampling method. To generate accurate estimates for relatively rare indicators, such as “tail lesions”, a higher percentage of animals per pen would have to be sampled than recommended in the WQP. The present study, in which all piglets were assessed during the farm visit, confirms this reasoning.

In summary, out of 10 indicators originating from the WQP in this study, “wounds on the body”, “lameness”, “skin condition”, “hernias”, and “tail lesions” achieved acceptable to good results in TRR in rearing piglets. However, this only applies to category 1, as the prevalence was too low for the calculation of the TRR in category 2 for the three-point scale indicators.

### Individual-level indicators selected from other sources

Considering the prevalence from the selected indicators originated from other sources, it is not possible to give a conclusive assessment for the following IIN: *“*abdominal wall”, “eye alteration”, “neurological disorders”, and “reddened eyes”. The remaining other-indicators, which are discussed further in the following, are “tail length”, “tail posture”, “normal behavior”, “back posture”, “claw alterations”, and “ear lesions”.

“Normal behavior” achieved acceptable results with regard to TRR in both comparisons (age class, batch). It can, thus, be recommended for use. However, “back posture” only achieved acceptable results in the age class comparison, but not in the batch-wise comparisons. This is most likely due to the fact that in the batch-wise comparison, other animals were assessed. These may have had a different health status (“real differences”, first reason for a low TRR). Moreover, “back posture” was prone to ­influences by the observer entering the pen, i.e., TRR may be enhanced by an evaluation from outside the pen. Moreover, clearer categorization might be helpful, as it was not easy even for the same observer to assess which smallest change in the “back posture” had to be assessed as category 1, especially in a big pen with moving animals. Overall, “back posture” is an important indicator of early disease detection and thus should be implemented during daily routine checks as well as during farms’ self-assessments. However, the usefulness in longer term welfare assessments needs further studies with clearer classification criteria and an evaluation from outside the pen.

Acceptable to good reliability results were achieved for the three-point scale indicator “tail length” in the present study. The good results for category 1 (docked tails/partial loss) are probably due to the docked tails of the piglets from Farm B. It would be advisable to verify the results on undocked piglets. The different management decisions of tail docking should be taken into account in the interpretation of the results. Nevertheless, the indicator can be recommended for the use in on-farm welfare assessment protocols. These results are confirmed by the good interobserver reliability in the study by Pfeifer et al. (2019).

“Tail posture” achieved acceptable results for the reliability parameters at least for the age class comparisons, whereby again the docked tails on Farm B limit the results of the present study, as assessing of tail posture on docked tails is difficult. Moreover, the observer entered the pen for the assessment thereby changing the tail posture of the animals. This could be the reason for the low prevalence of “tail posture”, which ranged between 0.00% and 8.10% for the age classes, compared to a prevalence of 0.30% to 53.0% in the study of Drexl et al. (2022). It is noticeable that almost no exact agreement (no acceptable or good values for the LoA) was achieved. However, still, given the acceptable reliability, “tail posture” can be used for self-monitoring on-farm. This supports the findings of Pfeifer et al. (2019), who suggested the limitation that “tail posture” should be evaluated by the same observer.

“Claw alterations” had an acceptable to good reliability in terms of RS, ICC, and for LoA in the present study. The indicator had already been proven with regard to its TRR in the study by Friedrich et al. (2020) in sows with similar results. However, in the same study, the authors detected a low interobserver reliability, which may be problematic with regard to longer term welfare assessment. As to date, no knowledge about the interobserver reliability of this indicator on rearing piglets exists, thus it can be recommended for use especially with regard to self-monitoring on farm.

The TRR of “ear lesions” can be interpreted as sufficient, which is in accordance with the good results for the interobserver reliability of Pfeifer et al. (2019).

To summarize, out of 10 indicators originating from other sources than the WQP in this study, the following 6 achieved acceptable to good results in TRR in rearing piglets: “tail length”, “tail posture”, “normal behavior”, “back posture”, “claw alterations”, and “ear lesions”.

### The four welfare principles

Of all the selected WQP-indicators considered in this study, the best results in terms of TRR in rearing piglets were achieved by: “sneezing” as well as “BO” from the PIN and “lameness” as well as “tail lesions” from the IIN. Thus, it would not be possible to cover the four welfare principles in the rearing period with the WQP-indicators, especially in the principles “good feeding”, “good housing” and partly “good health” problems occur. The reasons for a low TRR of these WQP-indicators (time of occurrence of the health problem, age of the animals, etc.) have already been discussed in detail. With the inclusion of the additional indicators derived from other sources than the WQP, a comprehensive assessment of the different welfare principles becomes more possible, especially for the welfare principle “good health” (“ear lesions”, “normal behavior”, “back posture” etc.). However, no adequate indicators for “good feeding” and “good housing” could be identified, which may also be due to the management, the localization, and climate management of the farms. For the welfare principle “appropriate behavior”, the “BO” could be complemented by “tail posture” and replace the “HAR”. Given the fact that this study chose to include comprehensive welfare indicators for rearing piglets as described in literature up to date, this is important knowledge for welfare assessment of rearing piglets in the future.

## Conclusion

The aim of the present study was to assess whether indicators for growing pigs can be used for rearing piglets, as has been recommended so far. To clarify this question, the TRR of the indicators were evaluated and it was determined whether the TRR is influenced by the group of assessed animals (batch) or the age of the assessed piglets. To do this, 28 indicators were selected from different self-monitoring protocols and tested regarding their TRR. Nine out of eighteen indicators (and for four indicators in category 2) originating from the WQP had a prevalence close to zero, which made a calculation for statistical parameters meaningless. The four welfare principles, which together cover the multidimensional field of animal welfare and on this basis the WQP, cannot be adequately assessed with the remaining WQP-indicators (low prevalence for rearing piglets in this study). In particular, there are problems with the welfare principles of “good feeding”, of “good housing”, and partly “good health”. To detect injuries or diseases (welfare principle “good health”), which are often at an early stage in rearing piglets, “back posture”, “ear lesions”, and “normal behavior” as indicators originated from other sources could be included in the WQP because they achieved acceptable to good results for the TRR. These indicators can complement the WQP-indicators with acceptable to good TRR, such as “BO”, “HAR”, “lameness”, “sneezing”, “tail lesions”, “wounds on the body”. However, no adequate indicators for “good feeding” and “good housing” could be identified.

## Abbreviations:

BO: behavioral observations
HAR: human–animal-relationship test
ICC: intraclass correlation co-efficient
IIN: individual-level indicators
LoA: limits of agreement
PIN: pen-level indicators
RS: Spearman’s rank correlation co-efficient
TRR: test–retest reliability
WQP: Welfare Quality protocols
